# The impact of COVID-19 pandemic on treatment delay and short-term neurological functional prognosis for acute ischemic stroke during the lockdown period

**DOI:** 10.3389/fneur.2022.998758

**Published:** 2022-10-20

**Authors:** Shiyuan Gu, Jie Li, Huachao Shen, Zhengze Dai, Yongjie Bai, Shuai Zhang, Hongyi Zhao, Suiyun Zhou, Yan Yu, Wuzhuang Tang

**Affiliations:** ^1^Department of Neurology, Affiliated Yixing Hospital of Jiangsu University, Yixing, China; ^2^Department of Neurology, Jinling Hospital, Medical School of Nanjing University, Nanjing, China; ^3^Department of Neurology, The Fourth Affiliated Hospital of Nanjing Medical University, Nanjing Pukou Hospital, Nanjing, China; ^4^Department of Neurology, First Affiliated Hospital, College of Clinical Medicine, Henan University of Science and Technology, Luoyang, China; ^5^Department of Neurology, Affiliated Hospital of Yangzhou University, Yangzhou, China; ^6^Department of Neurology, No. 984 Hospital of PLA, Beijing, China

**Keywords:** acute stroke, COVID-19, intravenous thrombolysis, mechanical thrombectomy, treatment delay

## Abstract

**Background:**

Preventive strategies implemented during the COVID-19 pandemic may negatively influence the management of patients with acute ischemic stroke (AIS). Nowadays, studies have demonstrated that the pandemic has led to a delay in treatment among patients with AIS. Whether this delay contributes to meaningful short-term outcome differences warranted further exploration.

**Objective:**

The objective of this study was to evaluate the impacts of the COVID-19 pandemic on treatment delay and short-term outcomes of patients with AIS treated with IVT and MT.

**Methods:**

Patients admitted before (from 11/1/2019 to 1/31/2020) and during the COVID-19 pandemic (from 2/1/2020 to 3/31/2020) were screened for collecting sociodemographic data, medical history information, and symptom onset status, and comparing the effect of treatment delay. The patients treated with IVT or MT were compared for delay time and neurological outcomes. Multivariable logistic regression was used to estimate the effect of treatment delay on short-term neurological prognosis.

**Results:**

In this study, 358 patients receiving IVT were included. DTN time increased from 50 min (IQR 40–75) before to 65 min (IQR 48–84), *p* = 0.048. 266 patients receiving MT were included. The DTP was 120 (112–148) min vs. 160 (125-199) min before and during the pandemic, *p* = 0.002. Patients with stroke during the pandemic had delays in treatment due to the need for additional PPE (*p* < 0.001), COVID-19 screening processes (*p* < 0.001), multidisciplinary consultation (*p* < 0.001), and chest CT scans (*p* < 0.001). Compared with pre-COVID-19, during the pandemic, patients had a higher likelihood of spontaneous intracranial hemorrhage after IVT (OR: 1.10; 95% CI, 1.03–1.30) and a lower likelihood of mRS scores 0–2 at discharge (OR: 0.90; 95% CI, 0.78–0.99). In logistic regression analysis, high NIHSS score at admission, increasing age, worse pre-admission mRS, large vessel occlusion, admission during the lockdown period, and low mTICI grade after MT were associated with an mRS ≥ 3.

**Conclusion:**

The COVID-19 pandemic has had remarkable impacts on the management of AIS. The pandemic might exacerbate certain time delays and play a significant role in early adverse outcomes in patients with AIS.

## Introduction

The likelihood of acute ischemic stroke (AIS) patients with large vessel occlusion (LVO) to receive emergency care, such as endovascular mechanical thrombectomy (MT) or intravenous thrombolysis (IVT), is extremely time-dependent (1). This is partially influenced by the effectiveness of pre-hospital care and the sufficiency of hospital resources. However, the impact of the pandemic is perhaps inevitable. Since the pandemic breakout, there has been a noticeably lower rate of thrombolysis and thrombectomy in patients with AIS, as documented by various facilities (2–5). Meanwhile, several articles have reported that the COVID-19 outbreak was linked to delays in the treatment of patients with AIS (6–8). Speculative explanations for these delays include the disruption of medical services cause by the pandemic, the anxiety of patients over contracting SARS-CoV-2, psychological stress brought on by the pandemic, and associated lockdowns (8, 9).

Hospitals must take the required precautions due to the outbreak escalation to prevent the simultaneous spread of the SARS-CoV-2 virus to patients and medical staff (10). According to reports, some medical facilities increased their precautionary procedures in responding to the pandemic, thus leading to longer delays in diagnosis and treatment (11), resulting in poor outcomes. Even during the pandemic, thrombolysis and thrombectomy should be administered to patients with AIS without any delay to reduce mortality and morbidity (12). With the escalation of the pandemic, the preventive measures around the world have also been upgraded accordingly. During the pandemic, China had launched several control measures to gradually reduce COVID-19 transmission. Recent studies have also shown a decrease in stroke admissions during the pandemic, but data on emergency stroke management and treatment outcomes are still limited (13). Therefore, we performed a multicenter retrospective study to compare treatment processes and clinical outcomes of patients with AIS who underwent IVT and MT before and after the pandemic outbreak to evaluate the impact of the pandemic on the processes and outcomes of IVT and MT performed in patients with AIS.

## Methods

### Study design and patient population

This study was part of an ongoing program for analyzing the COVID-19 pandemic in managing patients with stroke. The current study was a retrospective analysis of prospectively collected data. A total of six tertiary hospitals with comprehensive stroke centers were included in this study, four of which are in Jiangsu Province, namely, Jinling Clinical College of Nanjing Medical University, Affiliated Yixing Hospital of Jiangsu University, The Fourth Affiliated Hospital of Nanjing Medical University, and The Affiliated Hospital of Yangzhou University. The First Affiliated Hospital and College of Clinical Medicine of Henan University of Science and Technology is located in Henan Province, and NO 984 Hospital of PLA is in Beijing. On 31 January 2020, the Chinese government announced several nationwide strategies for preventing the COVID-19 pandemic. Patients with AIS diagnosed from 1 December 2019 to 31 January 2020 (pre-COVID-19), and those diagnosed from 1 February 2020 to 31 March 2020 (post-COVID-19) were compared in this study. AIS was diagnosed based on clinical symptoms and computed tomography or magnetic resonance imaging. Patients who reached the hospitals within 7 days after stroke onset were included. Socioeconomic status, medical history, stroke symptoms, National Institutes of Health Stroke Scale (NIHSS) score, Alberta Stroke Program Early CT Score (ASPECTS), modified thrombolysis in cerebral infarction (mTICI) score before discharge, onset-to-door (OTD) time defined as the time from onset to hospital arrival, door-to-needle (DTN) time defined as the time from hospital arrival to initiation of thrombolysis, door-to-puncture (DTP) time defined as the time from hospital arrival to groin puncture, and post-treatment NIHSS scores were reviewed and analyzed. All participants and their relatives provided written informed consent, and the study was approved by the ethics committees of the participating hospitals. The reporting of this study conformed to the STROBE statement (14). The emergency department staff were equipped with adequate personal protective equipment (PPE). Nucleic acid tests, body temperature measurements, inquiries about recent travel, complete blood count checks, and chest CT scans were all part of the COVID-19 screening process. Patients with definite fever or respiratory symptoms, as well as those whose routine chest CT scans suggested COVID-19 imaging, would need to undergo in-hospital multidisciplinary consultation. According to an expert consensus on the stroke emergency map during the epidemic of coronavirus disease 2019 (15), hospitals involved in the study had a 24/7 on-call COVID-19 expert group (associate chief physician and above), including respiratory physicians, infection physicians, critical care physicians, imaging physicians, emergency medicine physicians, respiratory nurses, and critical care nurses, to closely coordinate with the stroke green channel, responsible for the consultation of patients with suspected COVID-19. We would consult patients with suspected COVID-19 acute stroke (mainly by video consultation) to clarify the diagnosis and guide the clinical treatment and protection strategy. Patients were triaged by multidisciplinary consultation based on the results of these screenings. In brief, COVID-19 nucleic acid-negative patients requiring MT were treated in routine standard operating procedures. Suspected positive patients were treated in a specialized operating room with the highest level of protection and transferred to a specialized isolation ward after surgery. Patients with AIS undergoing MT procedures were generally recommended to undergo local anesthesia, unless the patients were irritable and uncooperative.

The primary outcome of this trial was the mRS score at discharge after IVT or MT. Safety outcomes included intracerebral hemorrhage (ICH), arterial perforation, subarachnoid hemorrhage (SAH), and arterial entrapment (a complication of entrapment of the internal carotid artery, the vertebrobasilar artery entrapment, and the large intracranial vessels).

All variables with a *p* < 0.1 were then entered into the multivariable logistic regression model—influencing factors for short-term clinical poor outcomes. For patients treated with IVT, variables such as age, sex, stroke etiology, pre-admission mRS, NIHSS at admission, stroke history, hypertension, diabetes, hyperlipidemia, atrial fibrillation, and coronary heart disease were included in the full model. For patients with MT treatment, mTICI 2b-3 was also included in the model. In this study, variables such as smoking, alcohol drinking, anterior circulation, solitary, daytime onset, and residence were removed from the full to final models.

### Statistical analysis

Continuous variables were expressed as mean ± standard deviation or median and interquartile range (IQR), as appropriate. Categorical variables were presented as frequency and percentage. Continuous variables with normal distribution were compared using Student's *t*-test. The χ2 and Fisher's exact tests were used for comparing categorical values. Multivariable stepwise logistic regression was used to determine influencing factors for short-term clinical poor outcomes (mRS ≥ 3) among all the patients with AIS enrolled in this study. A two-sided *p*-value of < 0.05 was deemed statistically significant. All statistical analyses were performed using SPSS 25.

## Results

**Figure 1:**
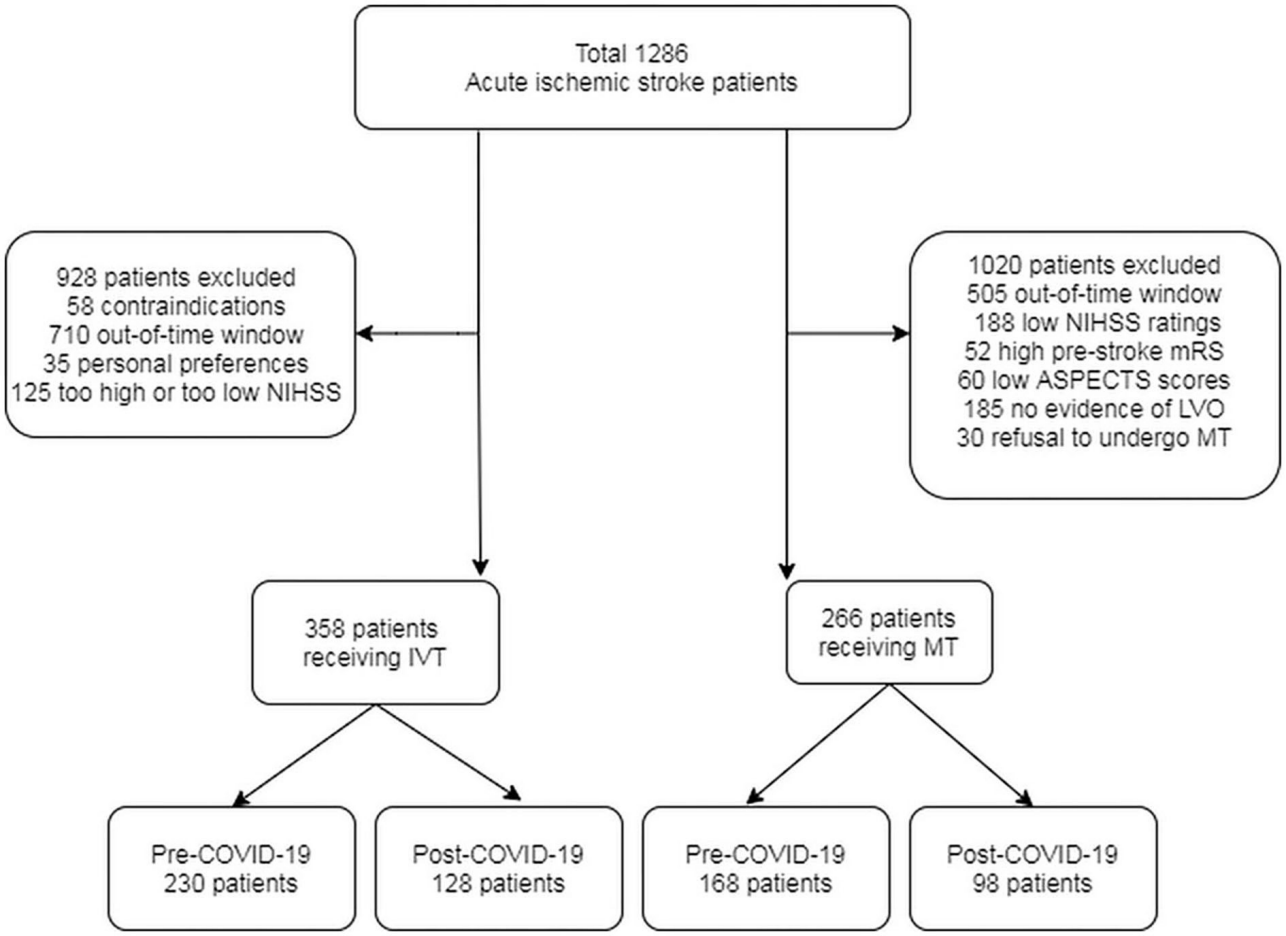
The flow chart of acute stroke patients receiving IVT or MT treatment during pre- and post-COVID-19 period.

In this study, a total of 1,286 patients with AIS were enrolled, among which 928 were ultimately not treated with thrombolysis due to contraindications, out-of-time window, personal preferences, and either too high or too low NIHSS ratings (Figure 1). Finally, 358 patients receiving IVT were included (Table 1), with 230 (64.2%) in the pre-COVID-19 group and 128 (35.7%) in the post-COVID-19 group. No discernible baseline differences were found between the two groups. All the post-COVID-19 group patients underwent additional screening, such as a chest CT scan and a multidisciplinary evaluation. In the pre-COVID-19 group, the median admission NIHSS score (IQR) was 9 (6–13), but in the post-COVID-19 group, it was 11 (7–15). According to expectations, the patients admitted during the COVID-19 period exhibited more severe symptoms (*p* = 0.020). OTD time increased from 68 min (interquartile range [IQR] 40–111) before to 85 min (IQR 45–127) after the COVID-19 pandemic (*p* = 0.001). DTN time increased from 50 min (IQR 40–75) before to 65 min (IQR 48–84) after the COVID-19 pandemic (*p* = 0.048). Compared with the pre-COVID-19 group, post-COVID-19 patients with IVT treatment had a higher rate of symptomatic intracranial hemorrhage (sICH) (*p* = 0.043). No significant differences were found in early neurological improvement, in-hospital mortality, mRS score (0-2) pre-admission, and at discharge between the two groups (Table 1).

**Table 1:** Characteristics of acute ischemic stroke patients with intravenous thrombolysis treatment before and during the COVID-19 pandemic.

**Characteristics**	**Pre-COVID-19** ** (*n* = 230)**	**Post-COVID-19** ** (*n* = 128)**	***p* value**
Age, year, mean	71.5 ± 10.1	70.9 ± 10.9	0.422
Male, sex, *n* (%)	138 (60.0)	75 (58.6)	0.737
Education, *n* (%)			0.511
Elementary education	69 (30.0)	35 (27.3)	
Secondary education	121 (52.6)	76 (59.4)	
Higher education	40 (17.4)	17 (13.3)	
Solitary, *n* (%)	75 (32.6)	46 (35.9)	0.110
Residence, *n* (%)			0.128
Urban	142 (61.7)	80 (62.5)	
Rural	88 (38.3)	48 (37.5)	
Daytime onset, *n* (%)	188 (81.7)	110 (85.9)	**0.033**
Stroke etiology *n* (%)			0.540
Large artery atherosclerosis	147 (63.8)	84 (65.6)	
Small vessel disease	31 (13.5)	17 (13.3)	
Cardioembolism	48 (20.9)	25 (19.5)	
Other demonstrated cause	2 (0.9)	1 (0.8)	
Undetermined cause	2 (0.9)	1 (0.8)	
NIHSS, median (IQR)	9 (6-13)	11 (7-15)	**0.020**
Stroke history, *n* (%)	46 (20.0)	29 (22.7)	0.179
Hypertension, *n* (%)	155 (67.4)	88 (68.8)	0.208
Diabetes, *n* (%)	95 (41.3)	55 (42.9)	0.225
Hyperlipidemia, *n* (%)	71 (30.9)	41 (32.0)	0.750
Atrial fibrillation, *n* (%)	35 (15.2)	18 (14.1)	0.209
Coronary heart disease, *n* (%)	47 (20.4)	28 (21.9)	0.177
Smoking, *n* (%)	104 (45.2)	56 (43.8)	0.353
Alcohol drinking, *n* (%)	88 (38.3)	48 (37.5)	0.501
OTD, min, median (IQR)	68 (40–111)	85 (45–127)	**0.050**
DTN, median (IQR), min, *n* (%)	50 (40–75)	65 (48–84)	**0.046**
In-hospital mortality, *n* (%)	11 (4.8)	7 (5.5)	0.220
sICH, *n* (%)	15 (6.5)	11 (8.6)	**0.043**
Pre-admission mRS (0–2), *n* (%)	125 (54.3)	62 (47.4)	0.072
mRS (0–2) at discharge, *n* (%)	138 (60.0)	72 (56.3)	0.180

IQR, interquartile range; NIHSS, National Institutes of Health Stroke Scale; mRS, modified Rankin Scale; sICH, symptomatic intracranial hemorrhage. Bold values are significant at *p* < 0.05.

Of the patients enrolled, 1020 of them were excluded for out-of-time window, low NIHSS scores, high pre-stroke mRS scores, low ASPECTS, no evidence of large artery occlusion, or refusal to undergo MT (Figure 1). A total of 266 patients with intracranial large artery occlusion were finally included (Table 2); of these, 168 (63.2%) were in the pre-COVID-19 group and 98 (36.8%) in the post-COVID-19 group. Compared with the pre-COVID-19 group, post-COVID-19 group patients had a higher NIHSS score [12 (9–17) vs. 10 (7–15), *p* = 0.042], and a lower percentage of pre-admission mRS (0–2) [68 (37.5%) vs. 29 (29.6%), *p* = 0.011], but no significant differences were found in the remaining baseline characteristics, including ASPECTS and the percentage of anterior circulation. The OTD time was 72 (38–118) min vs. 87 (41–136) min in the pre- and post-COVID-19 groups, *p* = 0.012. The DTP time was 120 (112–148) min vs. 160 (125–199) min before and after the pandemic, *p* = 0.002. No significant difference was found in the mTICI 2b-3 scores of the two groups (86.2 vs. 82.4 %, *p* = 0.088). Adverse events, including sICH, were not significantly different between the two groups (Table 2).

**Table 2:** Characteristics of acute ischemic stroke patients with mechanical thrombectomy treatment before and after the COVID-19 pandemic.

**Characteristics**	**Pre-COVID-19** ** (*n* = 168)**	**Post-COVID-19** ** (*n* = 98)**	***p* value**
Age, year, mean	71.7 ± 10.4	70.6 ± 10.6	0.320
Male, sex, *n* (%)	86 (51.2)	52 (52.0)	0.107
Education, *n* (%)			0.721
Elementary education	50 (29.7)	27 (27.5)	
Secondary education	92 (54.8)	56 (57.1)	
Higher education	26 (15.5)	15 (15.3)	
Solitary, *n* (%)	42 (25.0)	26 (26.5)	0.310
Residence, *n* (%)			0.225
Urban	101 (60.1)	61 (62.2)	
Rural	67 (39.9)	37 (37.8)	
Daytime onset, *n* (%)	142 (84.5)	82 (83.7)	0.110
Stroke etiology *n* (%)			0.788
Large artery atherosclerosis	152 (90.5)	90 (91.8)	
Small vessel disease	0	0	
Cardioembolism	16 (9.5)	8 (8.2)	
Other demonstrated cause	0	0	
Undetermined cause	0	0	
NIHSS, median (IQR)	10 (7–15)	12 (9–17)	**0.042**
Stroke history, *n* (%)	35 (20.8)	22 (22.4)	0.189
Hypertension, *n* (%)	116 (69.0)	66 (67.3)	0.207
Diabetes, *n* (%)	68 (40.5)	41 (41.8)	0.525
Hyperlipidemia, *n* (%)	52 (31.0)	32 (32.6)	0.750
Atrial fibrillation, *n* (%)	29 (17.2)	17 (17.4)	0.929
Coronary heart disease, *n* (%)	36 (21.4)	24 (24.5)	0.097
Smoking, *n* (%)	84 (50.0)	51 (52.0)	0.251
Alcohol drinking, *n* (%)	75 (44.6)	47 (47.9)	0.081
OTD, min, median (IQR)	72 (38–118)	87 (41–136)	**0.012**
DTP, min, median (IQR)	120 (112–148)	160 (125–199)	**0.002**
Puncture to reperfusion time, min, median (IQR)	41 (29–54)	35 (27–47)	0.120
Onset to reperfusion time, min, median (IQR)	250 (178–330)	288 (190–385)	**0.045**
ASPECTS, (IQR)	9 (8–10)	9 (8–10)	1.000
Anterior circulation, *n* (%)	121 (72.0)	70 (71.4)	0.504
mTICI2b-3, *n* (%)	142 (84.5)	81 (82.7)	0.188
Adverse events, *n* (%)	46 (27.4)	29 (29.6)	0.220
Pre-admission mRS (0–2), *n* (%)	68 (37.5)	29 (29.6)	0.011
mRS score 0–2 at discharge, *n* (%)	98 (58.3)	52 (53.1)	**0.050**

IQR, interquartile range; NIHSS, National Institutes of Health Stroke Scale; mRS, modified Rankin Scale; ASPECTS, Alberta Stroke Program Early CT Score; mTICI, modified thrombolysis in cerebral infarction. Bold values are significant at *p* < 0.05.

The post-COVID-19 group underwent a substantial delay for patients receiving IVT or MT due to the need for additional PPE (*p* < 0.001), COVID-19 screening processes (*p* < 0.001), multidisciplinary consultation (*p* < 0.001), and chest CT scans (*p* < 0.001). No significant differences were found in the proportion of patients receiving intravenous antihypertensive medication, family hesitancy about therapy, or hypoglycemia between the two groups before IVT or MT (Tables 3, 4).

**Table 3:** Influencing factors for delayed intravenous thrombolysis treatment.

**Variables**	**Pre-COVID-19** ** (*n* = 230)**	**Post-COVID-19** ** (*n* = 128)**	***p* value**
Need for additional PPE, *n* (%)	0	47 (36.7)	**<0.001**
COVID-19 screening processes, *n* (%)	0	128 (100)	**<0.001**
Multidisciplinary consultation, *n* (%)	13 (5.7)	24 (18.8)	**<0.001**
Hypertension requiring aggressive control with IV medications, *n* (%)	18 (7.8)	12 (9.3)	0.133
Initial refuse, *n* (%)	45 (19.6)	24 (18.8)	0.067
Blood glucose < 50 mg/dl, seizures or major metabolic disorders, *n* (%)	11 (4.8)	5 (3.9)	0.115
Equipment-Related Delay, *n* (%)	6 (2.6)	6 (4.7)	**<0.001**
Need for chest CT scans, *n* (%)	87 (37.8)	128 (100)	**<0.001**
Other, *n* (%)	23 (10.0)	12 (9.4)	0.335

PPE, personal protective equipment. Bold values are significant at *p* < 0.05.

**Table 4:** Influencing factors for delayed mechanical thrombectomy treatment.

**Variables**	**Pre-COVID-19** ** (n = 168)**	**Post-COVID-19** ** (n = 98)**	***p* value**
Need for additional PPE, *n* (%)	0	36 (36.7)	**<0.001**
COVID-19 screening processes, *n* (%)	0	98 (100)	**<0.001**
Multidisciplinary consultation, *n* (%)	11 (6.5)	19 (19.4)	**<0.001**
Hypertension requiring aggressive control with IV medications, *n* (%)	30 (17.8)	20 (20.4)	**0.013**
Initial refuse, *n* (%)	29 (17.2)	16 (16.3)	0.564
Need for chest CT scans, *n* (%)	52 (31.0)	98 (100)	**<0.001**
Care team unable to determine eligibility, *n* (%)	10 (5.9)	5 (5.1)	0.079
Equipment-Related Delay, *n* (%)	10 (5.9)	6 (5.1)	0.079
Need for additional imaging, *n* (%)	52 (31.0)	29 (29.6)	0.106
Catheter Lab Not Available, *n* (%)	18 (10.7)	11 (11.2)	0.098
Other, *n* (%)	29 (17.2)	18 (18.3)	0.260

PPE, personal protective equipment. Bold values are significant at *p* < 0.05.

Table 5 presents the potential influencing factors for the prognosis of AIS by multivariate logistic regression analysis. Compared with the patients before the pandemic, the patients during the COVID-19 lockdown period had an odds ratio (OR) of 1.10 (95% confidence interval [CI], 1.03–1.30) for spontaneous intracranial hemorrhage, and an OR of 0.90 (95% CI, 0.78–0.99) for mRS scores 0–2 at discharge, whereas no significant differences were found in the proportion of lower extremity venous thromboembolism (VTE) or pulmonary embolism (PE) during hospitalization and discharge disposition (home, inpatient rehabilitation).

**Table 5:** Association of the COVID-19 pandemic with outcomes among patients with AIS.

	**OR (95% CI)**	*p* value^*^
sICH among IV alteplase patients	1.10 (1.03–1.30)	**0.050**
VTE or PE during hospitalization	1.21 (0.83–2.10)	0.202
Discharge mRS 0–2	0.90 (0.78–0.99)	**0.028**
Discharge to inpatient rehabilitation facility	0.88 (0.73–1.39)	0.321
Discharge to home	2.10 (0.73–3.00)	0.520

* Regression models compare outcomes in patients during the COVID-19 period to those before the pandemic. Models are adjusted for patient demographics, clinical characteristics, medical history, and hospital characteristics.

OR, odds ratio; CI, confidence interval; NIHSS, National Institutes of Health Stroke Scale; sICH, symptomatic intracerebral hemorrhage within 36 h of thrombolysis; VTE, venous thromboembolism; PE, pulmonary embolism. Bold values are significant at *p* < 0.05.

In the current study, we found increasing age, OR: 1.81 (1.18–2.92), *p* = 0.005; worse pre-admission mRS, OR: 1.30 (1.15–1.48), *p* = 0.010; higher NIHSS score at admission, OR: 2.84 (1.45–4.8), *p* < 0.001; large vessel occlusion, OR: 2.02 (1.32–3.05), *p* < 0.001; admission during the lockdown period, OR: 1.22 (1.02–1.34), *p* = 0.050; mTICI grade 2b-3 after MT, OR: 0.44 (0.25–0.67), *p* < 0.001, to be significantly associated with poor outcomes in AIS (mRS ≥ 3) by logistic regression, whereas sex, history of stroke, hypertension, diabetes, hyperlipidemia, atrial fibrillation, and coronary artery disease were not (Table 6).

**Table 6:** Multivariate logistic regression analysis of influencing factors for short-term clinical poor outcomes (mRS ≥ 3).

**Variables**	**OR**	**95% CI**	***p* value**
Increasing age	1.81	1.18-2.92	**0.005**
Sex (female)	0.90	0.81-1.22	0.330
pre-admission mRS	1.30	1.15-1.48	**0.010**
Higher NIHSS at admission	2.84	1.45-4.88	**<0.001**
Large vessel occlusion	2.02	1.32-3.05	**<0.001**
COVID-19 pandemic	1.22	1.02-1.34	**0.050**
Stroke history	1.31	0.88-2.01	0.434
Hypertension	1.01	0.92-1.24	0.886
Diabetes	1.28	0.96-1.20	0.132
Hyperlipidemia	1.19	0.90-1.18	0.675
Atrial fibrillation	1.29	0.85-1.45	0.366
Coronary heart disease	0.99	0.87-1.25	0.278
mTICI 2b-3	0.44	0.25-0.67	**<0.001**

OR, odds ratio; CI, confidence interval; NIHSS, National Institutes of Health Stroke Scale; mRS, modified Rankin Scale; ASPECTS, Alberta Stroke Program Early CT Score; mTICI, modified thrombolysis in cerebral infarction. Bold values are significant at *p* < 0.05.

## Discussion

In this retrospective study with focus on the COVID-19 lockdown period, we evaluated the impact of the pandemic on treatment delays and short-term clinical outcomes in patients with AIS. In this study, DTN time and DTP time were significantly longer during the COVID-19 period than during the pre-COVID-19 period. In our previous study, we found that the onset-to-door time was significantly prolonged during the COVID-19 pandemic compared with that in the pre-pandemic period (16). In this study, the need for additional PPE, viral nucleic acid testing, and chest CT scans were the main causes of in-hospital delay during the pandemic. PPE contributed to the delay in treatment times mainly due to the shortage or unavailability including the filtering facepiece respirators and gowns in the early lockdown period. In addition, the pandemic in itself was an independent risk factor for treatment delay and short-term unfavorable outcomes in patients with treated stroke. A large registry study involving 55,296 patients with AIS showed that in-hospital mortality was higher in patients with delayed thrombolytic therapy and that treatment delay was associated with poor clinical outcomes (17). According to a previous study, patients admitted during the COVID-19 pandemic experienced pre- and in-hospital treatment delays to varying extents (16).

Recent research has shown that even minor delays might have a negative impact on clinical outcomes in the short term (18). In the current study, the COVID-19 pandemic played a significant role in early adverse outcomes in patients with AIS. This effect is mainly attributed to the pandemic, which exacerbated certain time delays. The proportion of VTE or PE during hospitalization was not significantly associated with the COVID-19 pandemic partly due to the relatively short hospital stay in this study. In addition, a marginal increase in in-hospital mortality was noted among patients with AIS during the pandemic, which may have been due to the greater severity of stroke (19). Many patients with mild to moderate stroke avoided hospital admissions during the lockdown period, as indicated by the reports from some countries, which showed a 50–80% reduction in acute stroke admissions (20). The increased NIHSS score in the post-COVID-19 period partially supports this proposition. A similar pattern of delay in seeking medical care due to the fear of being infected within the hospital was observed during the Ebola epidemic in West Africa (21).

Our study also indicated that admission during the pandemic was an independent risk factor for short-term mortality and other adverse outcomes. It is crucial to identify and examine specific stroke workflows to improve stroke reperfusion rates and clinical outcomes. According to a previous study, inadequate imaging techniques may result in delays (22). However, this finding was unnoticed in our study and some other studies (23, 24). Kansagra et al. (25) reported a 39% decrease in the use of stroke imaging during the early COVID-19 pandemic period. Among these phases, delays from imaging to thrombolysis were the main factor responsible for the overall delay. Therefore, future research concentrating on imaging flow may be crucial to achieving a reduction in treatment delays in the ongoing pandemic. The findings of the current investigation (26) indicate that the prolongation of DTN time and DTP time can have a negative impact on short-term outcomes.

The strength of this study was that it included a large number of patients with non-COVID strokes based on real-world data. While previous studies from China for the most part focused on Wuhan, which was the initial area of the COVID-19 outbreak, we collected data from three provinces away from Wuhan, which could reflect the impact of the pandemic on stroke management outside the epicenter. Another strength of our study was that we examined how the pandemic affected treatment delays and clinical outcomes in patients with treated strokes and also identified the pandemic as an independent risk factor for poor short-term prognosis in AIS.

## Limitation

A main limitation of this study was its retrospective, observational design, which made it prone to selection bias. Second, because COVID-19 -infected patients were not included in this study, we could not analyze any potential detrimental effects of the virus on patient prognosis. Third was the lack of data on the other changes that could have contributed to the delay such as adjustments made to triage protocols and availability of staff due to extremely busy emergency personnel during the earliest stages of the pandemic. Furthermore, the time of treatment delay in bridging therapy, which means patients received both thrombolysis and MT treatment, was not specifically collected by some centers, so the potential detrimental effects on the bridging therapy were not analyzed. In addition, the long-term effects of treatment delay on the 90-day functional outcomes of patients with AIS were not examined in the current study. Therefore, prospective multicenter studies with sizable sample numbers are needed to clarify the aforementioned findings.

## Conclusion

In-hospital delays during the COVID-19 pandemic negatively impacted the treatment of non-COVID strokes in China. Given that anti-COVID-19 measures are evolving into medical norms, stroke centers need to evaluate local practice patterns to optimize the management processes and lessen the impact of the pandemic on clinical outcomes in patients with AIS.

## Data availability statement

The raw data supporting the conclusions of this article will be made available by the authors, without undue reservation.

## Author contributions

SG, JL, and ZD: study design, interpretation of results, and manuscript drafting. SG, YB, JL, HZ, and HS: study design and interpretation of results. YY, ZD, YB, JL, HS, HZ, and SZ: data collection. WT, YY, SZ, JL, YB, ZD, HS, and SG: study design, statistical analysis, and critical revision of the manuscript. SG and WT: interpretation of results, critical revision of the manuscript, has full access to all of the data in the study, took responsibility for the integrity of the data, and the accuracy of the data analysis. All authors contributed to the article and approved the submitted version.

## Funding

The work was supported by the Foundation of Jiangsu Province of China (Z2021001), the Health Institute of Wuxi (M202157), and Top Talent Support Program for young and middle-aged people of Wuxi (Yixing) Health Committee (BJ2020108).

## Conflict of interest

The authors declare that the research was conducted in the absence of any commercial or financial relationships that could be construed as a potential conflict of interest.

## Publisher's note

All claims expressed in this article are solely those of the authors and do not necessarily represent those of their affiliated organizations, or those of the publisher, the editors and the reviewers. Any product that may be evaluated in this article, or claim that may be made by its manufacturer, is not guaranteed or endorsed by the publisher.
